# The administration of *Enterococcus faecium* SF68 counteracts compositional shifts in the gut microbiota of diet-induced obese mice

**DOI:** 10.3389/fmicb.2022.1054097

**Published:** 2022-12-16

**Authors:** Adelaide Panattoni, Marco Calvigioni, Laura Benvenuti, Vanessa D’Antongiovanni, Carolina Pellegrini, Clelia Di Salvo, Diletta Mazzantini, Francesco Celandroni, Matteo Fornai, Luca Antonioli, Emilia Ghelardi

**Affiliations:** ^1^Department of Translational Research and New Technologies in Medicine and Surgery, University of Pisa, Pisa, Italy; ^2^Department of Clinical and Experimental Medicine, University of Pisa, Pisa, Italy

**Keywords:** probiotics, gut microbiota, *Enterococcus*, obesity, high-fat diet, ileum

## Abstract

Microorganisms with probiotic properties are eliciting an increasing interest as coadjuvants in the prevention and treatment of obesity through modulation of the gut microbiota. In this study, a probiotic formulation based on *Enterococcus faecium* SF68 was administered to mice fed with a high-fat diet (HFD) to evaluate its efficacy in reducing body mass gain and in modulating the intestinal bacterial composition. Both stool and ileum samples were collected from untreated and treated mice and absolute abundances of specific *taxa* constituting the gut microbial *consortium* were evaluated. SF68 administration significantly reduced the HFD-induced weight gain. In these animals, the microbial gut composition shifted toward an enrichment in microbes positively correlated with mucus thickness, lower inflammation, lower glycemia levels, and SCFA production (i.e., *Bifidobacterium*, *Akkermansia*, and *Faecalibacterium*), as well as a depletion in bacterial phyla having a key role in obesity (i.e., *Firmicutes*, *Proteobacteria*). Our results demonstrate the efficacy of *E. faecium* SF68 in adjusting the composition of the dysbiotic microbiota of HFD-fed animals, thus ameliorating clinical conditions and exerting anti-obesity effects.

## Introduction

The administration of probiotics, “live microorganisms which, when administered in adequate amounts, confer a health benefit on the host” (Food and Agricultural Organization of the United Nations and World Health Organization, 2001), is often effective in ameliorating the symptoms of a wide variety of clinical disorders in humans. These include antibiotic-associated diarrhea (Mekonnen et al., 2020), infectious and *Clostridioides difficile*-associated diarrhea (Sánchez et al., 2017), irritable bowel syndrome (Moayyedi et al., 2010), type 2 diabetes (Ejtahed et al., 2011), inflammatory bowel diseases [e.g., ulcerative colitis (Ganji-Arjenaki and Rafieian-Kopaei, 2018) and obesity (Tenorio-Jiménez et al., 2020)], through countless but poorly investigated mechanisms of action.

Exploiting probiotic microorganisms as coadjuvants in the treatment or prevention of obesity is of clinical relevance. Obesity is a pathological condition that consists of abnormal or excessive fat accumulation, which in turn often drives to other chronic disorders (Cerdó et al., 2019), such as metabolic syndrome, insulin-resistance, and cardiovascular pathologies (Frasca et al., 2017). Moreover, this condition is typically associated with several other biomarkers. Due to aberrant intestinal permeability and mucus thickness, obese patients display high levels of lipopolysaccharide in the bloodstream (Green et al., 2020), a condition known as metabolic endotoxemia, which can lead to a constant state of systemic low-grade inflammation (Cani et al., 2007a). This prolonged inflammatory state contributes to insulin-resistance, triggering the onset of type-2 diabetes and metabolic issues (Cani et al., 2007b). Several studies showed how different probiotic strains were able to improve obesity-related conditions (Yadav et al., 2013; Bagarolli et al., 2017; Ejtahed et al., 2019). By restoring mucus thickness and tight junctions’ expression or regulating the production of different cytokines, probiotics can improve the gut barrier integrity and modulate immune functions (Cerdó et al., 2019), as well as stimulate the secretion of satiety-related hormones through the production of short chain fatty acids (SCFAs; Da Silva et al., 2021). Besides these specific mechanisms of action, probiotics can also modulate the gut microbiota composition, thus triggering cascading effects that affect the entire organism (Azad et al., 2018). Considering the severe alteration in the fecal microbiota observed in obese patients, with higher levels of *Firmicutes* (Abenavoli et al., 2019) and *Actinobacteria* (Bibbò et al., 2016) and lower abundances of *Bifidobacterium* and *Akkermansia* (Gérard, 2016) than normal-weight subjects, the administration of probiotics may be considered a potential therapeutic approach aimed at restoring eubiosis of the intestinal flora and counteracting the clinical scenario of obesity.

Previous studies demonstrated the efficacy of selected *Enterococcus* strains in a variety of conditions (i.e., intestinal dysbiosis, metabolic syndrome, high levels of serum cholesterol and triglycerides, and inflammation) due to their recognized ability to secrete propionate and butyrate, hydrolyze bile salts, inhibit the transcription of pro-inflammatory mediators, and modulate the composition of the gut microbiota (Allen et al., 2010; Avram-Hananel et al., 2010; Tarasova et al., 2010; Zhang et al., 2017; Greuter et al., 2020; Mishra and Ghosh, 2020; Huang et al., 2021). Considering the overall positive effects demonstrated in these studies, enterococci seem to be promising candidates for the prevention and treatment of obesity and amelioration of other obesity-related complications.

In this study, *Enterococcus faecium* SF68 was orally administered to mice fed with a high-fat diet (HFD) to evaluate its efficacy in reducing body mass gain, modulating the gut microbiota composition, and restoring a well-balanced intestinal flora. Quantitative analyses were performed to obtain information on the bacterial composition found in both fecal and ileal tissue samples, thus elucidating the role of *E. faecium* SF68 in the interaction with intestinal residing microorganisms and toward amelioration of the obesity condition.

## Materials and methods

### Experimental design and probiotic administration

**Figure 1:**
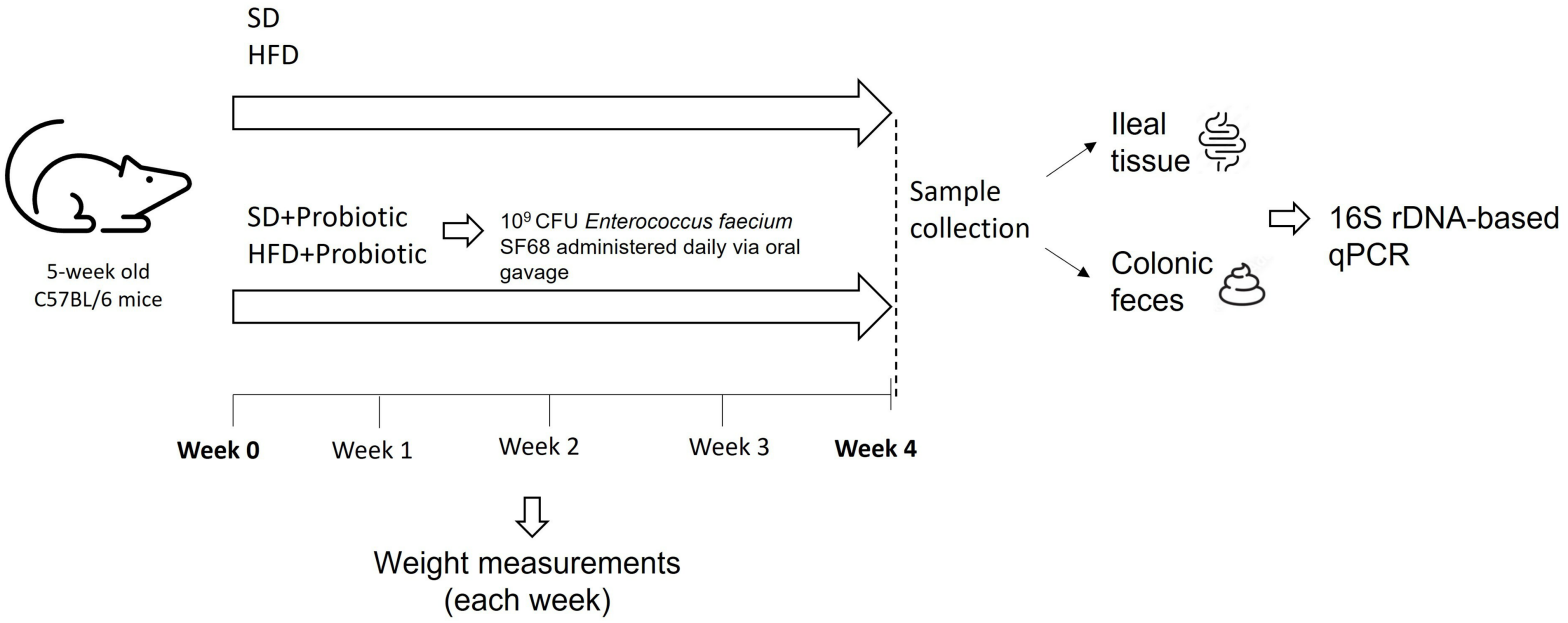
Schematic representation of the experimental design.

In this study, C57BL/6 mice were used as an animal model. Animals were supplied by ENVIGO s.r.l (Italy). Mice were five-week-old and weighed 20 g upon reception. For the duration of the study, mice were housed in cages on a 12-h light cycle, with a room temperature of 22–24°C and 50–60% humidity. Animals were allowed free access to food and water *ad libitum*. They were handled and cared following the European Community Council Directive 2010/63/UE, transposed by the Italian Government. The study was approved by the Italian Ministry of Health (authorization 955/2018-PR). Mice were randomly divided in 4 groups, each constituted of 5 to 10 mice (Figure 1). Two of the four groups were fed with a standard diet (SD, TD.2018), which provided 58% kcal as carbohydrates, 24% kcal as proteins, and 18% kcal as fats (3.1 kcal/g), while the remaining groups were fed with a high-fat diet (HFD, TD.06414), which provided 21.4% kcal as carbohydrates, 18.3% kcal as proteins, and 60.8% kcal as fats (5.1 kcal/g). Both dietary regimes were purchased from ENVIGO s.r.l. Animals belonging to one of the two SD groups were treated with a probiotic formulation based on *Enterococcus faecium* SF68 (SD + SF68), and the same procedure was performed for animals fed with the HFD (HFD + SF68). *E. faecium* SF68 (Cerbios-Pharma SA, Switzerland) is currently available in worldwide commercialized probiotic formulations as a drug. To administer the probiotic formulation, 10^9^ CFU of *E. faecium* SF68 were resuspended in 0.15 ml of methocel 3%. Animals received the product daily *via* oral gavage for 4 weeks. After this period, animals were weighed, then anaesthetized and sacrificed to collect feces (withdrawn directly from the sigmoid colon) and ileal samples (tissue and mucus layer deprived of luminal content). All samples were stored separately at −80°C until use.

### Genomic DNA extraction from feces and ileal tissue

To perform genomic DNA extraction from fecal and ileal samples, QIAmp PowerFecal Pro DNA Kit (QIAGEN, Germany) and QIAmp DNA Mini Kit (QIAGEN) were used, respectively. The extraction procedure was performed following the manufacturer’s protocol. Fecal and ileal samples to extract weighed 15 and 25 mg, respectively. DNA concentration was calculated by measuring the optical density at 260 nm (OD260) and DNA purity was estimated by determining the OD260/ OD280 ratio with Nanodrop Lite Spectrophotometer (Thermo Fisher Scientific, United States).

### Real-Time quantitative PCR

To assess the absolute abundances of total bacteria, and of the main phyla (i.e., *Firmicutes*, *Bacteroidetes*, *Actinobacteria*, *Proteobacteria*) and genera (i.e., *Bacteroides*, *Bifidobacterium*, *Faecalibacterium*, *Lactobacillus*, *Prevotella*) present in both fecal and ileal samples, 16S rRNA gene-based Real-Time qPCR reactions were performed. Quantification of *Akkermansia muciniphila* abundance in ileal samples was also carried out. Primer pairs used in this study, able to specifically anneal to phylum- or genus- specific regions of the gene encoding 16S rRNA, are listed in Tables 1, 2. A degenerate primer pair, used to determine the total bacteria abundance, is also reported (Table 1). PCR reactions were performed using the CFX96 Real-Time System (BioRad, United States), and absolute quantifications were extrapolated using the CFX Manager Software (Biorad). Bacterial quantifications were established using calibration curves, obtained with serial 10-fold dilutions of external standards with known concentration ranging from 10^2^ to 10^8^ DNA copies/μl. Reactions were carried out in duplicate in a 96-wells plate with a final reaction volume of 20 μl, containing 1 μl of 2.5 ng/μl DNA template, 10 μl of Luna Universal qPCR Master Mix (New England BioLabs, United States), 0.5 μl of each primer (0.25 μM), and 8 μl of sterile water. The amplification conditions were as follows: an initial denaturation step at 95°C for 1 min, followed by 45 cycles of denaturation at 95°C for 15 s, annealing at primers’ optimal temperature for 30 s (Tables 1, 2), and extension at 72°C for 10 s (Biagini et al., 2020). To check the amplification specificity, a melting curve analysis was carried out after PCR reactions by increasing the annealing temperature from 65 to 95°C.

**Table 1:** Primer pairs used in this study for the quantification of total bacterial load and microbial phyla.

Investigated bacterial group	Primer name and sequence (5′-3′)	Amplicon length (bp)	Annealing temperature (°C)	References
All bacteria	F: ACTCCTACGGGAGGCAGCAG	200	60	Biagini et al. (2020)
	R: ATTACCGCGGCTGCTGG			
*Firmicutes*	F: ATGTGGTTTAATTCGAAGCA	126	62	Biagini et al. (2020)
	R: AGCTGACGACAACCATGCAC			
*Bacteroidetes*	F: CATGTGGTTTAATTCGATGAT	126	62	Biagini et al. (2020)
	R: AGCTGACGACAACCATGCAG			
*Actinobacteria*	F: CGCGGCCTATCAGCTTGTTG	600	65	Stach et al. (2003)
	R: CCGTACTCCCCAGGCGGGG			
*Proteobacteria*	F: CATGACGTTACCCGCAGAAGAAG	195	63	Biagini et al. (2020)
	R: CTCTACGAGACTCAAGCTTGC			

**Table 2:** Primer pairs used in this study for the quantification of microbial genera and *Akkermansia muciniphila*.

Investigated bacterial group	Primer name and sequence (5′-3′)	Amplicon length (bp)	Annealing temperature (°C)	References
*Akkermansia muciniphila*	F: CAGCACGTGAAGGTGGGGAC	329	50	Collado et al. (2007)
	R: CCTTGCGGTTGGCTTCAGAT			
*Bacteroides*	F: GAGAGGAAGGTCCCCCAC	106	60	Kim et al. (2017)
	R: CGCTACTTGGCTGGTTCAG			
*Bifidobacterium*	F: CTCCTGGAAACGGGTGG	550	55	Matsuki et al. (2002)
	R: GGTGTTCTTCCCGATATCTACA			
*Faecalibacterium*	F: GGAGGAAGAAGGTCTTCGG	248	50	Kim et al. (2017)
	R: AATTCCGCCTACCTCTGCACT			
*Lactobacillus*	F: GAGGCAGCAGTAGGGAATCTTC	126	65	Kim et al. (2017)
	R: GGCCAGTTACTACCTCTATCCTTCTTC			
*Prevotella*	F: GGTTCTGAGAGGAAGGTCCCC	121	60	Kim et al. (2017)
	R: TCCTGCACGCTACTTGGCTG			

### Statistical analysis

All data are expressed as mean ± standard deviation. Statistical analyses were carried out with GraphPad Prism 8 (GraphPad Software Inc., United States). For all comparisons (SD vs. HFD; SD vs. SD + SF68; HFD vs. HFD + SF68) statistical significance was set at a *p*-value of 0.05 and assessed using Student *t*-tests for unpaired data.

## Results

### Weight variations in mice

**Figure 2:**
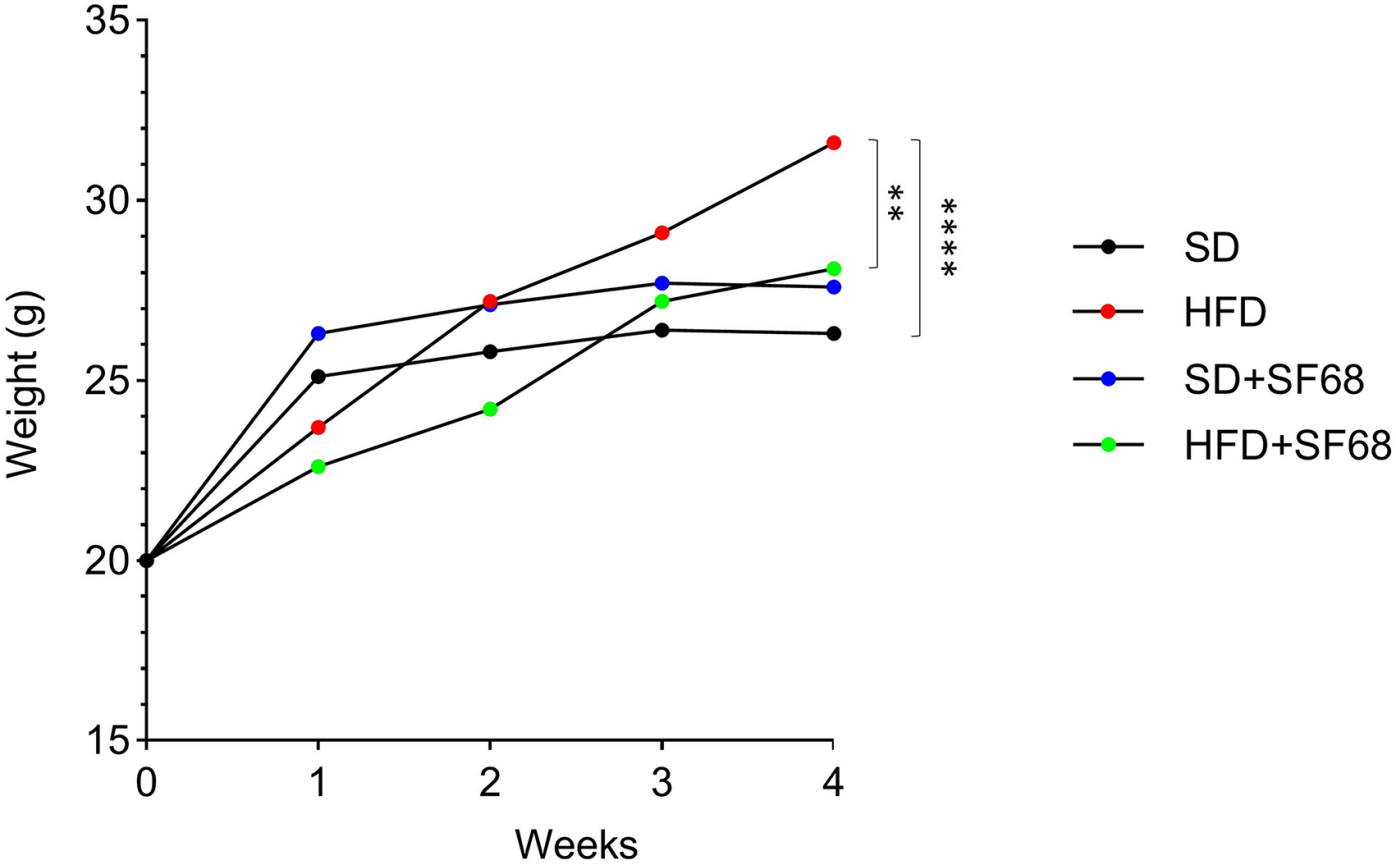
Weight variations in groups SD, SD + SF68, HFD, and HFD + SF68 registered weekly up to 4 weeks. **p < 0.01; ****p < 0.0001.

Mice fed with HFD displayed a significant increase in body weight after 3 weeks (29.10 ± 2.56 g, +10.2%, *p* = 0.0038) and 4 weeks (31.60 ± 2.91 g, +20.2%, *p* < 0.0001) of dietary regimen compared to those fed with SD (3 W, 26.40 ± 0.84 g; 4 W: 26.30 ± 0.95 g), thus confirming the efficacy of the dietary regimen in setting up an obesity model (Figure 2). Interestingly, while no statistically significant weight differences were evidenced between SD and SD + SF68 during the entire experimental protocol (*p* > 0.05), mice receiving the probiotic formulation with the concomitant HFD gained less weight than the HFD control group at 2, 3, and 4 weeks (2 W, −12.4%, *p* = 0.0006; 3 W: −7.0%, *p* = 0.0376; 4 W: −16.2%, *p* = 0.0017).

### Microbiota composition in SD and HFD mice

**Figure 3:**
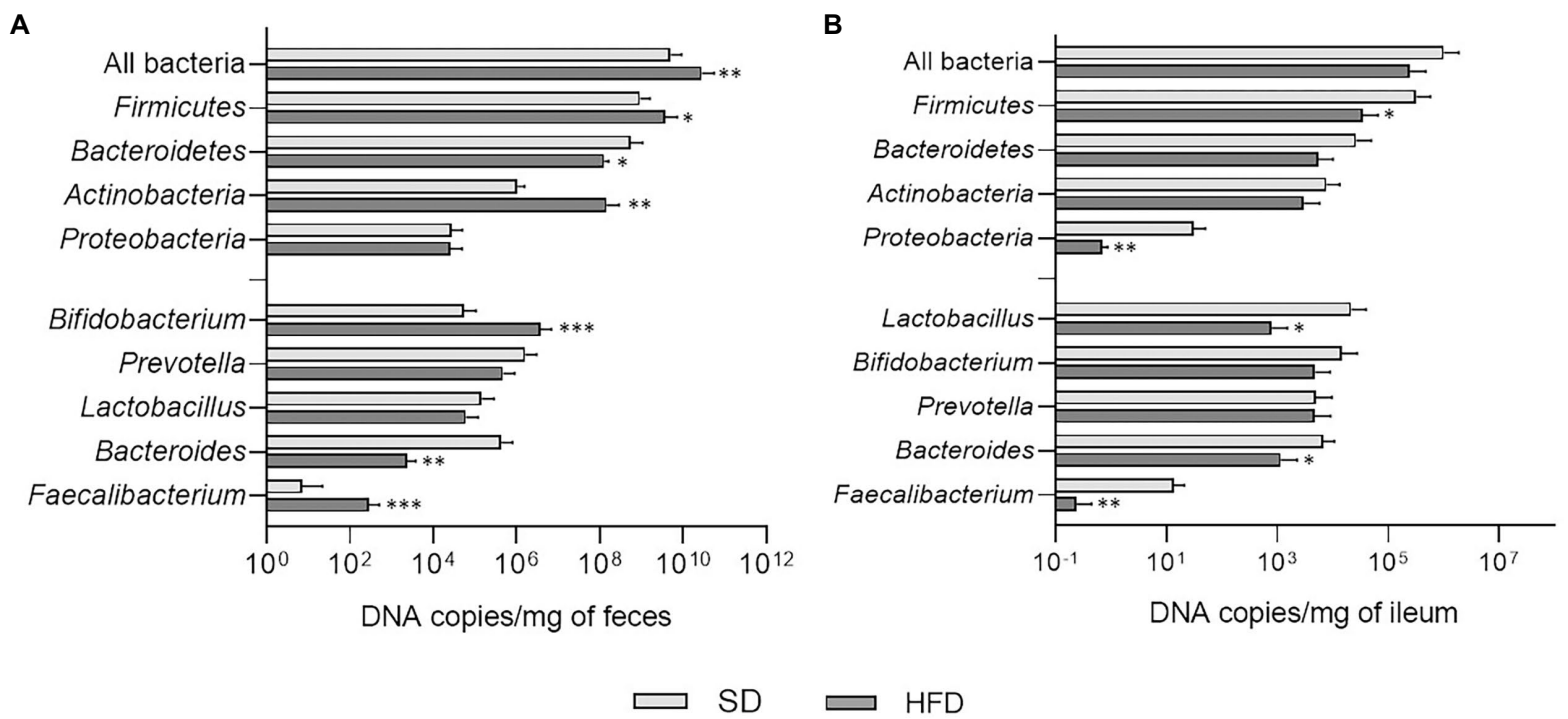
**(A)** Absolute abundances of total bacterial load, phyla, and genera in fecal samples of mice belonging to SD and HFD groups. **(B)** Absolute abundances of total bacterial load, phyla, and genera in ileal samples of mice belonging to SD and HFD groups. *p < 0.05; **p < 0.01; ***p < 0.001.

In fecal samples of HFD-fed mice, significant increases were observed in the total amount of bacteria (*p* = 0.0095), *Firmicutes* (*p* = 0.0162), and *Actinobacteria* (*p* = 0.0024), while *Bacteroidetes* abundance resulted lower (*p* = 0.0395; Figure 3A). At genus level, these animals displayed an increase in *Bifidobacterium* (*p* = 0.0007) and *Faecalibacterium* (*p* = 0.0003), while a decrease was registered in *Bacteroides* (*p* = 0.0037; Figure 3A). The analysis of ileal samples provided different results. *Firmicutes* (*p* = 0.0397) and *Proteobacteria* (*p* = 0.0067) amounts were significantly lower in mice fed with HFD compared to SD and reductions in the absolute abundance of *Lactobacillus* (*p* = 0.0363), *Bacteroides* (*p* = 0.0133), and *Faecalibacterium* (*p* = 0.0021) were also evident in this group of animals (Figure 3B).

### Fecal microbiota composition after probiotic treatment in SD- and HFD-fed mice

**Figure 4:**
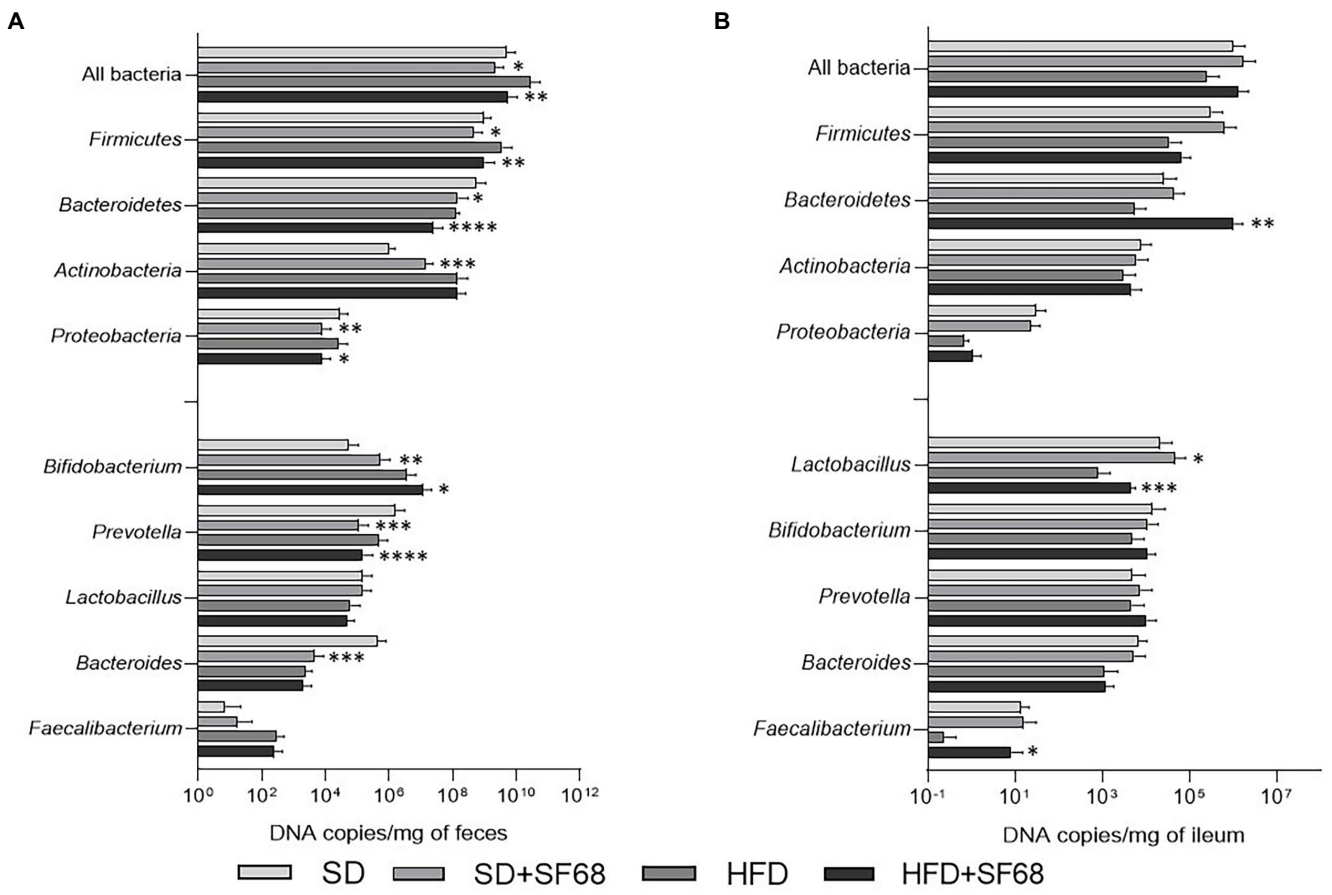
**(A)** Absolute abundances of total bacterial load, phyla, and genera in fecal samples of mice fed with SD or HFD with or without SF68 administration. **(B)** Absolute abundances of total bacterial load, phyla, and genera in ileal samples in both mice fed with SD or HFD with or without SF68 administration. Statistical comparisons were carried out between SD and SD + SF68, and HFD and HFD + SF68. *p < 0.05; **p < 0.01; ***p < 0.001; ****p < 0.0001.

The administration of *E. faecium* SF68 to SD- and HFD-fed mice caused a significant reduction of the fecal bacterial load (SD + SF68 vs. SD, *p* = 0.0275; HFD + SF68 vs. HFD, p = 0.0021), *Firmicutes* (SD + SF68 vs. SD, *p* = 0.0318; HFD + SF68 vs. HFD, *p* = 0.0063), *Bacteroidetes* (SD + SF68 vs. SD, *p* = 0.0199; HFD + SF68 vs. HFD, *p* < 0.0001) and *Proteobacteria* (SD + SF68 vs. SD, *p* = 0.0088; HFD + SF68 vs. HFD, *p* = 0.0203) compared to untreated animals (Figure 4A). An increase in the abundance of *Actinobacteria* was observed in normal weight mice that received the formulation (*p* = 0.0001). Both groups receiving the probiotic displayed a significant increase in the amount of bacteria belonging to the *Bifidobacterium* genus (SD + SF68 vs. SD, *p* = 0.0042; HFD + SF68 vs. HFD, *p* = 0.0364) and a reduction in *Prevotella* (SD + SF68 vs. SD, *p* = 0.0008; HFD + SF68 vs. HFD, p < 0.0001) in comparison with SD and HFD controls. A significant reduction was also evidenced in the amount of *Bacteroides* in SD + SF68 animals compared to SD controls (*p* = 0.0002).

### Ileal microbiota composition after probiotic treatment in SD- and HFD-fed mice

Absolute quantifications of bacteria in ileal samples revealed increased abundance of the phylum *Bacteroidetes* (*p* = 0.0092) and the genus *Faecalibacterium* (*p* = 0.0352) in HFD + SF68 group in comparison with HFD controls (Figure 4B). In addition, an increase in *Lactobacillus* was observed after probiotic treatment in both SD- and HFD-fed mice (SD + SF68 vs. SD, *p* = 0.0255; HFD + SF68 vs. HFD, *p* = 0.0004).

### *Akkermansia muciniphila* abundance in the ileal mucosa

**Figure 5:**
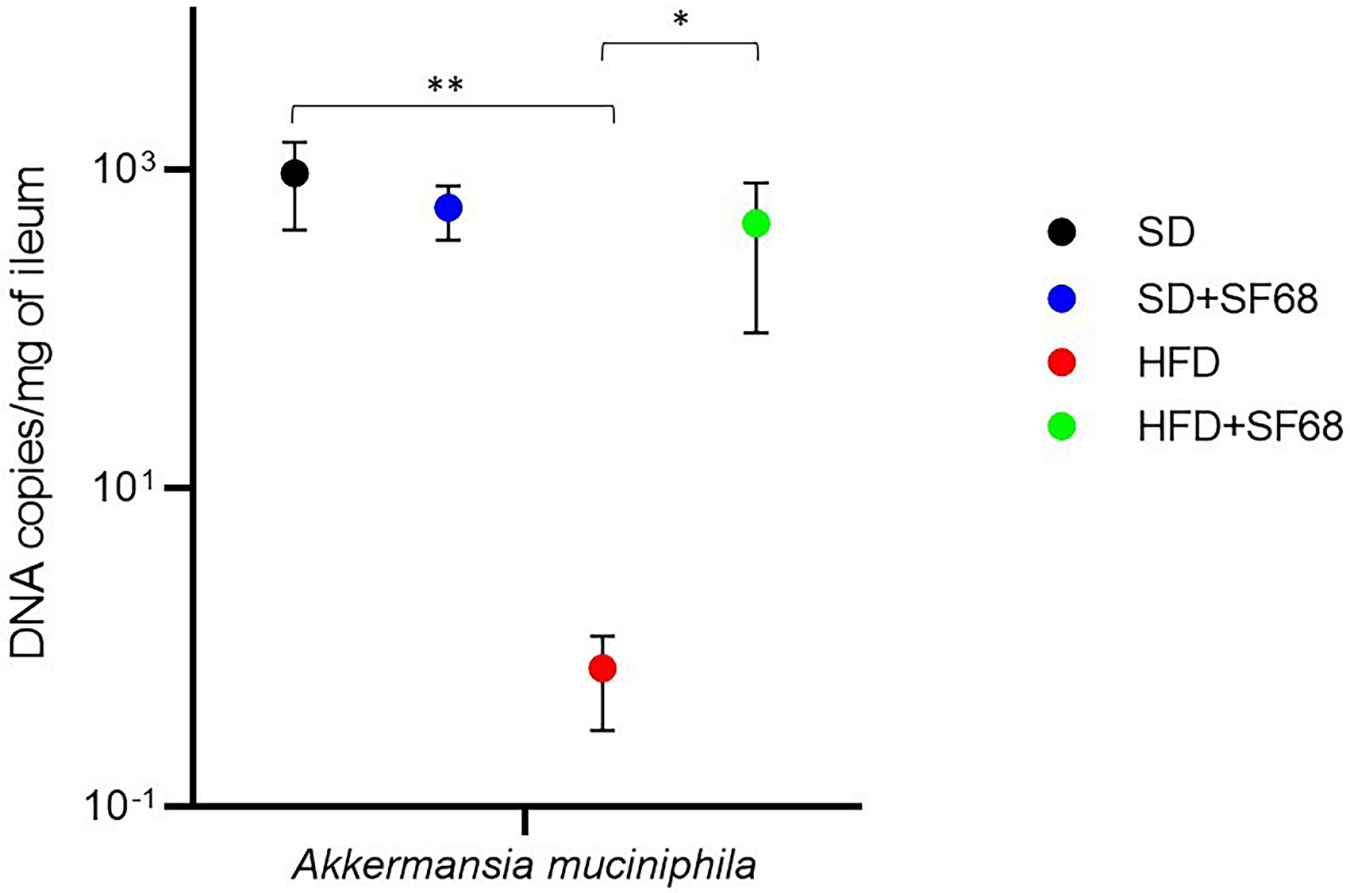
Variations in the absolute abundance of *Akkermansia muciniphila* in ileal tissues in both normal weight (SD) and obese (HFD) mice with or without SF68 administration. Statistical comparisons were carried out between SD and SD + SF68, and HFD and HFD + SF68. *p < 0.05; **p < 0.01.

The amount of *Akkermansia* was significantly lower in obese (HFD) than normal weight (SD) mice (*p* = 0.0026; Figure 5). Notably, a remarkable increase of this microorganism was revealed in HFD + SF68 mice (4.63 × 10^2^ ± 3.68 × 10^2^ of DNA copies/mg of ileum) compared to HFD-fed animals (7.45 × 10^−1^ ± 4.41 × 10^−1^ DNA copies/mg of ileum; *p* = 0.0341; Figure 5).

## Discussion

Mice are the most commonly used animals to study obesity after inducing this condition with either monogenic mutations or high-fat diets (Fuchs et al., 2018). High-fat diets have been shown to mimic more faithfully the overall metabolic and physiological alterations observed in obese individuals (Antonioli et al., 2020). For this reason, we selected this procedure to establish a proper experimental model of obesity and to obtain a more representative analysis of the gut microbiota, which was performed on both fecal and ileal samples. In fact, to investigate the entire gut microbiota composition, the analysis of feces alone might not be sufficient (Raoult and Henrissat, 2014; Li et al., 2017), since the microbial community associated with the host’s mucus, in particular that residing in the ileal tract, is not adequately represented in the stool. In addition, mucus-associated microorganisms of the upper intestine are actively involved in several physiological processes, such as immune regulation, inflammatory responses (Raoult, 2017), nutrient absorption, particularly of simple sugars (Raoult and Henrissat, 2014), and energy intake (Aron-Wisnewsky et al., 2012), which altogether play a key role in the development of obesity.

In our study, mice fed with HFD displayed a remarkable weight gain and substantial variations in their gut microbiota composition, which is usually found dysbiotic in obese individuals (Abenavoli et al., 2019). In particular, obese animals showed an increased fecal abundance of *Firmicutes* and *Actinobacteria*. Previous studies already showed an increase of members belonging to these phyla in fecal samples of obese subjects (Turnbaugh et al., 2006; Clarke et al., 2012; Grigor'eva, 2020). These samples were also demonstrated to contain a higher abundance of bacterial genes commonly associated with obesity and mainly derived from *Actinobacteria* and *Firmicutes* (Turnbaugh et al., 2009). The enrichment in *Firmicutes*, the most abundant phylum in both murine and human gut, has also been associated with increased nutrient absorption, thus suggesting *Firmicutes* as main microbial actors in obesity (Jumpertz et al., 2011).

In accordance with studies performed on obese subjects, which report low intestinal levels of *Bacteroides* being associated with visceral adiposity and obesity (Yin et al., 2022), we observed a reduction in the abundance of *Bacteroides* in both the feces and ileal mucosa of obese mice. We also detected a relevant decrease in *Lactobacillus* and *Faecalibacterium* in ileal samples of obese mice, consistently with previous studies regarding obesity, non-alcoholic fatty liver disease, and inflammatory bowel diseases (Sokol et al., 2009; Million et al., 2013; Lopez-Siles et al., 2018; Iino et al., 2019). Lactobacilli represent an important component of the microbial community residing in the human gut and exert a variety of beneficial properties (Li et al., 2017; Villmones et al., 2018), which make them the most common probiotics available in the worldwide market (Slattery et al., 2019). On the other hand, *F. prausnitzii*, the unique species belonging to the genus *Faecalibacterium* up to date, is considered a bioindicator for human health (Ferreira-Halder et al., 2017), exerting anti-inflammatory activities (Martín et al., 2015), producing butyrate (Louis and Flint, 2009), and improving the gut barrier function (Carlsson et al., 2013). We can therefore strongly presume that the reduction of *Lactobacillus* and *Faecalibacterium* observed in the HFD group hampered the preservation of a healthy condition in mice. The overall weight gain in HFD mice alongside with the variations we observed in both fecal and ileal microbiota allowed us to assert the robustness of our murine model of obesity.

Since specific *Enterococcus* strains display beneficial effects and satisfactory safety profiles when administered in a variety of pathological conditions in humans (Holzapfel et al., 2018), we wondered whether the oral administration of a probiotic formulation based on *E. faecium* SF68 was able to counteract the excessive weight gain associated with the obesogenic diet and prevent the shift to a dysbiotic gut microbiota. Some strains of *Enterococcus* are known for their ability to lower serum cholesterol (i.e., *E. faecium* CRL 183, M74, and WEFA23) (Foulquié Moreno et al., 2006; Franz et al., 2011; Zhang et al., 2017), exert anti-inflammatory activities (i.e., *E. faecium* M-74 1, *E. durans* M4, and M5) (Avram-Hananel et al., 2010; Franz et al., 2011), increase insulin and leptin sensitivity and reduce adipocyte hypertrophy and fat accumulation (i.e., *E. faecalis* AG5; Mishra and Ghosh, 2020), and directly modulate the intestinal microbial composition (i.e., *E. faecium* L5, and R0026; Tarasova et al., 2010; Huang et al., 2021), thus showing endearing features in the management of obesity (De Carvalho Marchesin et al., 2018; Jiang et al., 2021). *E. faecium* SF68 is a well-known *Enterococcus* probiotic strains contained in commercialized formulations and has been proven to be safe and effective in the treatment of acute diarrhea (Allen et al., 2010; Greuter et al., 2020).

*E. faecium* SF68 administration to HFD-fed mice resulted in a remarkably lower weight gain. The concurrent variations observed in the intestinal microbiota were altogether encouraging. A reduction of fecal *Firmicutes* abundance was detected in both SD + SF68 and HFD + SF68 mice, thus suggesting an active role of the *Enterococcus* strain in the modulation of *Firmicutes* and in the preservation of an eubiotic state. Interestingly, in fecal samples of both SD- and HFD-fed mice a reduction in *Proteobacteria* after SF68 treatment was also detected. *Proteobacteria* are usually present in small percentages within a healthy gut microbiota. In fact, an association between high abundance of *Proteobacteria* and gut dysbiosis, which often occurs in case of metabolic disorders, intestinal inflammation, and obesity, has been proven (Shin et al., 2015; Litvak et al., 2017).

The abundance of *Bifidobacterium* notably increased in the fecal microbiota of SF68-treated SD- and HFD-fed mice. The correlation between the amount of bifidobacteria and obesity is well-established. A few studies reported higher *Bifidobacterium* abundances to be associated with normal weight conditions (Kalliomäki et al., 2008) and demonstrated that *Bifidobacterium* spp. negatively correlate with endotoxemia and positively correlate with improved glucose tolerance, reduced plasma levels of pro-inflammatory cytokines (Cani et al., 2007b), and higher mucus thickness (Moreira et al., 2012). Additionally, bifidobacteria have been proven to ameliorate obesity-related conditions, for example by reducing cholesterol and glucose levels (Yin et al., 2010; An et al., 2011).

A greater abundance of *Prevotella* in obese individuals (Stanislawski et al., 2019), as well as in patients with hypertension (Dan et al., 2019), has recently been reported. Our finding that *Prevotella* decreased in the fecal samples of both groups of mice after SF68 treatment supports the hypothesis that the reduction of this genus potentially corresponds to an improvement in mice’s overall wellbeing. The administration of SF68 also resulted in higher amounts of *Lactobacillus* and *Faecalibacterium* in the ileal mucosa of HFD-fed mice. Besides their aforementioned properties, microbes belonging to *Bifidobacterium*, *Lactobacillus*, and *Faecalibacterium* are additionally able to produce SCFAs. These compounds exert a variety of beneficial effects both at local and systemic level and can therefore ameliorate the clinical characteristics of obese patients (Canfora et al., 2015).

*A. muciniphila* is well-known for its inverse correlation with obesity, type 2 diabetes, and other metabolic conditions, such as hypercholesterolemia and fatty liver disease (Xu et al., 2020). Conversely, high levels of *Akkermansia* are associated with a better metabolic status, in particular with higher leptin sensitivity, lower fasting blood glucose and insulin resistance, and lower adipocyte size (Dao et al., 2016). Some of the molecular mechanisms of these associations are known. *A. muciniphila* is able to produce SCFAs as a result of mucin degradation (Ottman et al., 2017) and can counteract the onset of obesity by reducing appetite, energy intake, systemic inflammation, and blood pressure, and improve the gut barrier function (Le Blanc et al., 2017; Chambers et al., 2018). Similarly to results obtained in previous studies in which *A. muciniphila* was found to be decreased in obese mice (Everard et al., 2013), we assessed that the HFD-fed group displayed a lower abundance of this microorganism than animals receiving the standard diet. The administration of *E. faecium* SF68 to HFD-fed mice significantly increased *Akkermansia* levels, thus suggesting that the administration of such a probiotic could have a positive impact on the metabolism of mice fed with HFD.

In conclusion, relevant results were obtained when *E. faecium* SF68 was administered to mice fed with a high-fat diet, demonstrating its ability to positively influence the gut microbial community both at luminal and mucosal level and to counteract the weight gain that occurs with an obesogenic diet. Several variations in the microbiota composition were highlighted, such as the reduction in *Firmicutes* and *Proteobacteria* and the increase in *Bifidobacterium* in fecal samples, as well as an increase in *Faecalibacterium*, *Lactobacillus*, and *Akkermansia* in ileal samples. These findings evidence the potential of *E. faecium* SF68 as a promising approach for the co-treatment and prevention of obesity, also opening new perspectives in the study of the molecular mechanisms underlying its beneficial effect.

## Data availability statement

The datasets presented in this study can be found in online repositories. The names of the repository/repositories and accession number(s) can be found at: http://doi.org/10.17632/2syf8c7fsh.1.

## Ethics statement

The animal study was reviewed and approved by the Italian Ministry of Health (authorization 955/2018-PR).

## Author contributions

MF, LA, and EG: conception and design of the study, validation, formal analysis, and supervision. AP, MC, LB, VD’A, CP, CS, DM, and FC: methodology and investigation. AP and MC: writing – original draft preparation. AP, MC, LB, VD’A, CP, CS, DM, FC, MF, LA, and EG: writing – review and editing. All authors have read and agreed to the published version of the manuscript.

## Funding

This study was funded by grants from the University of Pisa (EG) and by Cerbios-Pharma SA (MF and LA).

## Conflict of interest

The authors declare that the research was conducted in the absence of any commercial or financial relationships that could be construed as a potential conflict of interest.

## Publisher’s note

All claims expressed in this article are solely those of the authors and do not necessarily represent those of their affiliated organizations, or those of the publisher, the editors and the reviewers. Any product that may be evaluated in this article, or claim that may be made by its manufacturer, is not guaranteed or endorsed by the publisher.
